# The Chinese Version of Rochester Participatory Decision-Making Scale (RPAD): Reliability and Validity

**DOI:** 10.1155/2020/4343815

**Published:** 2020-12-12

**Authors:** Xufang Guo, Huan Liu, Yian Shih, Cheng Wang, Chuan Gao, Zhong He

**Affiliations:** ^1^School of Humanities and Social Sciences, Peking Union Medical College, Chinese Academy of Medical Science, Beijing, China; ^2^School of Nursing, Peking University, Beijing, China; ^3^Basic Medical College of Peking Union Medical College, Chinese Academy of Medical Science, Beijing, China

## Abstract

**Aim:**

This study aims to translate the Rochester Participatory Decision-Making Scale (RPAD) into the Chinese language and to test the reliability and validity of the Chinese version of the scale in the gynecological clinic.

**Methods:**

After obtaining the permission of the original author, the Brislin translation model was used to forward-translation and back-translation. Then, an expert group was set up to discuss this scale and result in cross-cultural adaptation. A convenient sampling method was used to select ten doctors working in the gynecological clinic of two top-three hospitals and 20 patients of each doctor. The Rochester Decision Participation Scale was used by the Chinese version for investigation.

**Results:**

The Chinese version of the Rochester Participatory Decision-Making Scale has a Cronbach's *α* coefficient of 0.604 for the total content reliability, the Spearman–Brown coefficient of half-reliability is 0.646, and the Guttman coefficient of half-reliability is 0.612. The retest reliability is 0.922. By exploratory factor analysis, the scale extracted three common factors, and the standard factor load corresponding to each entry is higher than 0.4.

**Conclusion:**

The reliability and validity of the Chinese version in the Rochester Participatory Decision-Making Scale are acceptable, which can be used to evaluate doctors “promotion of patients” participation in decision-making.

## 1. Introduction

As the medical model gradually shifts from disease-centered to patient-centered, the International Alliance of Patients' Organizations (IAOP) has also proposed five basic principles for achieving patient-centered healthcare: respect, choice, policy, access, and support, as well as information [1]. Therefore, the traditional model for doctors to formulate treatment strategies is challenging to meet the requirements of modern medicine, and adopting new decision-making methods has become a key measure for the development of health services.

The concept of shared decision-making (SDM) was formed in the 1990s. It is defined as “the process of joint selection between patients and their medical staff to help patients play an active role in making decisions about their healthcare” [1]. Specifically, the shared decision-making model is a collaborative process. Patients collaborate with doctors to make shared decisions through communication. During the process, the doctor will be asked to explain the different treatment options and their advantages and disadvantages to the patient and fully consider the existing scientific evidence, including the patient's values, goals, and preferences. Ultimately, the best treatment was chosen with the patients [2, 3]. Research shows that the model of shared decision-making can reduce internal conflicts between doctors and patients, reduce costs, maximize the function of the health service system, and strengthen people's ability to manage their illness [4, 5]. In order to ensure the quality of the shared decision-making model and allow patients to participate in decision-making, it is indispensable to use decision aids. Based on the decision-supporting framework and important components [6, 7], a variety of international decision-making aids (from patients' perspective [8], doctors' perspective [9], and evaluators' perspective [10, 11]) have been developed. Still, decision-making aids from the evaluators' perspective are few, and there is no Chinese version of the relevant scale. Therefore, this study aims to translate the Rochester Participation Decision-Making Scale (RPAD) and then test the reliability and validity based on the cross-cultural principle to provide an evaluation tool for the promotion of the shared decision-making model.

## 2. Background

Shields et al. developed the Rochester Decision Participation Scale in 2005, which was developed to evaluate physician communication behavior and to be used for physician training purposes [11]. In developing the RPAD, Shields and his team observed that some physician behaviors were performed entirely, whereas others were completed only partially. This finding led them to create a coding scheme for each item that gave a score of 0 for no evidence of the behavior, ½ for the partial presence of the behavior, and 1 for the full presence of the behavior. There are nine items in this scale. In the sixth item, the options range from “level of technicality or detail of the physician's and patient's language matches” to “clear mismatch between the technicality of physician's and patient's language,” which are counted as −½ points, ½ points, and 1 point, respectively. The options of the remaining 8 items range from “no evidence” to “patients actively participate in decision-making” and “doctors invite patients to participate,” with scores of 0, ½, and 1, respectively. The Chinese scale's score is the total score of each item, and the full score is 9. A higher score means that the doctor urges the patient to participate in decision-making better.

## 3. The Study

### 3.1. Aim

This study aims to translate the Rochester Participatory Decision-Making Scale (RPAD) into the Chinese language and to test the reliability and validity of the Chinese version of the scale in the gynecological clinic.

### 3.2. Methods

#### 3.2.1. Participants

The study was conducted in two top-level hospitals in China from December 2019 to April 2020. There are two sets of participants in this study: gynecologists and patients. We received ten gynecologists who were members of the two hospitals and 200 patients as convenience samples. The inclusion criteria for gynecologists are as follows: (a) qualified physician, (b) practice in gynecology endocrinology for more than 2 years, and (c) informed consent and voluntary participation in this study. Exclusion criteria were who are inappropriate from the researcher's perspective. The inclusion criteria of patients were who are 18–50 years of age, basic literacy, well communication, informed consent, and voluntarily participate in this study. Exclusion criteria were those who are suffering from neurological, psychiatric diseases, coronary heart disease, adrenal diseases, thrombotic diseases, other endocrine diseases, etc., inappropriate from the researcher's perspective.

#### 3.2.2. Instruments

*(1) General Information Questionnaire*. The collection of general information data for both the physicians and the patients is consistent with which the Rochester Participatory Decision-Making Scale includes. The physician questionnaire includes age, sex, and rural practice. The patient questionnaire includes age, length of the patient-physician relationship, and education.

*(2) Translation of RPAD*. After obtaining the consent of Professor Shields, the original author of the Rochester Participation Decision Scale, this study used Brislin's method of translation for forward-translation and back-translation. Forward-translation: two post-graduate students (XFG and HL) who major in medical English translated the scale from English to Chinese independently. Then, the researcher and a doctoral student in nursing compared the two translated versions and adjusted the objectionable items. The expert group which contains one doctor with a medical doctor degree, one professor with a nursing PhD degree, one doctor with a medical master's degree, one PhD student who has studied in the English-speaking country for three years, and two translators who discuss the combined RPAD in Chinese. Back-translation: a graduate student who has studied in the United States and not seen the original scale will translate the original Chinese RPAD back into English. One graduate student who majors in medical English was invited to compare the differences between the back-translated English version and the former scale items.

#### 3.2.3. Data Collection

After getting in touch with the gynecology departments of two hospitals, the members who are familiar with the questionnaire carry out the survey. The survey was conducted after the participants agreed, and the general questionnaire was retrieved and numbered on the spot. The Rochester Participation Decision Scale was used by the researchers who were trained in advance to score doctor-patient communication. The researchers recorded the communication process. 30 patients' name and time of the follow-up appointment were recorded for the retest. The same researcher scores doctor-patient communication again.

The evaluators scored with the RPAD Chinese version whenever the communication between doctor and patients started. Each item was given a score of 0 for no evidence of the behavior (did not discuss uncertainties in any way or did not give a clear description of the clinical problem), ½ for the partial presence of the behavior (did not totally discuss barriers to carrying out the treatment plan or physician language cannot totally match the patients'), and 1 for the full presence of the behavior (clarify agreement on the diagnosis and treatment). Besides, item 6 was given a score of −½ for no evidence.

#### 3.2.4. Ethical Considerations

All samples are obtained from two hospitals, and all participants have realized the research purpose and the research procedure, so they could decide whether they would like to participate. After they agreed to participate and signed informed consent, the researcher gave them the questionnaires.

#### 3.2.5. Statistical Analysis

All data were analyzed using the Statistical Package for the Social Sciences (SPSS; version 25.0). The internal consistency reliabilities of the RPAD Chinese version were estimated with Cronbach's coefficient. The cycle-to-cycle test-retest reliability was assessed with Pearson's correlation coefficient. The retest reliability was also assessed with Pearson's correlation coefficient. The content validity and structural validity (exploratory factor analysis and confirmatory factor analysis) were used to evaluate the validity of the RPAD Chinese version. *P* < 0.05 in the statistical results is statistically significant.

## 4. Results

### 4.1. Demographic Information on the Gynecologists and Patient

There are ten gynecologists and 200 patients involved in this study. The ten gynecologists are from the two top hospitals, and the practicing years in clinical are more than two years.  Table 1 shows more specific information about ten gynecologists. The 200 patients were 18 to 49 years old (32.26 ± 9.32). There are 37 (18.5%) patients who have less than nine years of education, 43 (21.5%) patients have 10–12 years of education, 32 (16%) patients graduated from 1 to 3 years college, and 81 (40.5%) patients graduated from 4 years college or graduate school. 103 (51.5%) patients were married, 71 (35.5%) patients were unmarried, and 23 (11.5%) patients were divorced or widowed.  Table 2 shows more specific information of 200 patients.

### 4.2. The Reliability of the RPAD Chinese Version

The internal consistency coefficient of the Chinese version of the Rochester Decision Participation Scale is 0.604 (standardized coefficient is 0.634), the Spearman–Brown coefficient of half-reliability is 0.646, and the Guttman coefficient of half-reliability is 0.612. 30 patients' name and time of the follow-up appointment were recorded for a retest. The retest reliability coefficient is 0.922, and the retest reliability of each item is between 0.503 and 0.952.

### 4.3. Content Validity of the RPAD Chinese Version

The Chinese version of the Rochester Participation Decision Scale was translated from the English version, which was developed by Professor Shields and achieved good reliability and validity. After that, experts in the clinical field, nursing field, and humanities field were invited to analyze and judge the content and range of the Chinese version scale. The result shows that the questionnaire can well represent the original scale.

### 4.4. The Exploratory Factor Analysis of the RPAD Chinese Version

Exploratory factor analysis was performed on the collected data. A principal components analysis produced three factors after varimax rotation, such as factor 1 (explanation and discussion), factor 2 (ask questions), and factor 3 (confirmation). The KMO was 0.518 (>0.5) and Bartlett's test of sphericity chi-square showed *P* < 0.001. The factors of each item are higher than 0.4 (Table 3).

## 5. Discussion

### 5.1. Acceptable Reliability of RPAD Chinese Version

The Rochester Participatory Decision-Making Scale (RPAD) was translated into the Chinese language and the reliability of the Chinese version was tested in the gynecological clinic. It is generally believed that if the reliability coefficients of scale are between 0.6 and 0.8, it indicates that the internal consistency is good. If it is higher than 0.8, it suggests that the internal consistency is excellent. The internal consistency coefficient of the RPAD Chinese version is 0.604, the Spearman–Brown coefficient of half-reliability is 0.646, and the Guttman coefficient of half-reliability is 0.612 which is lower than that reported for the RPAD English version [11]. This may be due to the different characteristics of the subjects. Firstly, the length of the patient-physician relationship is generally shorter, in which most subjects are less than one year. In comparison, in the professor Shields' study, more than half of the subjects had a doctor-patient relationship longer than five years. Then, there are 40 percent of patients having less than 12 years of education in this study as well as the number in the English version is only 7.1 percent.

30 patients' name and time of the follow-up appointment were recorded for a retest. The result of the statistical analysis showed the retest reliability coefficient is 0.922. The retest reliability of the item “The professional expression of the physician matches the patient's level of understanding” is low. One of the best reasons is that the patient and the doctor are more familiar with each other, compared with the first communication. Therefore, the communication process is slightly different from the first communication. In addition, there are only 30 retest cases that may have insufficient samples or the samples may not be representative.

In summary, the confidence of the RPAD Chinese version is reliable.

### 5.2. Acceptable Validity of RPAD Chinese Version

In this study, 74% of the physicians gave a clear description of the clinical problem, and almost 83% of the physicians tried to clarify agreement on the diagnosis and treatment plan which is similar to the result of the English version. Only 10% of patients were allowed to ask questions which is far less than the English version. Less than 5% of the time, physicians checked patients' understanding.

Based on the Braddock et al. scale and other literature on participatory decision-making, the scale items of the English version have face validity [12, 13]. After translating into Chinese, experts in the clinical medicine field, nursing field, and humanities field all affirm the content and range of the Chinese version scale which can prove to have good content validity.

After the exploratory factor analysis of the Chinese version of RPAD, three factors were extracted by principal component analysis. Factor 1 (explanation and discussion) was composed of 3 items: 1 (explain the clinical issues), 2 (discussion about the uncertain condition), and 3 (clarification of agreement). Factor 2 (ask questions) was composed of 3 items: 6 (match), 7 (any question), and 8 (open-ended questions). Factor 3 (confirmation) was composed of 3 items: 4 (recognize barriers with treatment plan), 5 (check patient's understanding of the treatment plan), and 9 (check the physician's understanding of the patient's view). The factors of each item are greater than 0.4 after principal component analysis, which means the validity of the RPAD Chinese version is acceptable.

### 5.3. Limitations

Through the analysis of demographic information on the physician and patient, this study found that the length of the patient-physician relationship is generally short. 48.5 percent of the samples have established a doctor-patient relationship for less than one year [11]. Therefore, it is inconvenient to build a shared decision-making model, and doctors urge patients to participate in decision-making. And it is hard for doctors to force patients to participate in decision-making.

In this study, the reliability of the Chinese version of the RPAD was tested in the gynecological clinic, which may be biased. In addition, the sample size of the retest reliability is too small, resulting in low retest reliability of some items. Therefore, the follow-up study will investigate different departments and exaggerate the sample size.

## 6. Conclusion

The reliability and validity of the Chinese version of RPAD are acceptable, and it can be used to evaluate physician communication behavior. However, the number of samples in this study is small, and the selected hospital department is single. Forming a scientific and rigorous scale requires further research based on the Chinese medical environment.

## Figures and Tables

**Table 1:** Characteristics of gynecologists in sample.

Number	Age	Sex	Family practitioner	Rural practice
1	57	Male	Yes	Yes
2	54	Male	No	Yes
3	55	Female	No	Yes
4	50	Female	Yes	Yes
5	48	Female	No	Yes
6	43	Female	No	Yes
7	40	Female	Yes	Yes
8	33	Female	Yes	Yes
9	28	Female	No	No
10	27	Female	No	No

**Table 2:** Characteristics of patients in sample.

Characteristic		Number	Percent (%)
Age	18–29	92	46
	30–39	56	28
	≥40	52	26
	Missing	0	0

Marital status	Unmarried	71	35.5
	Married	103	51.5
	Divorced or widowed	23	11.5
	Missing	3	1.5

Education	≤9 years	37	18.5
	10–12 years	43	21.5
	1–3 years college	32	16
	4 years college or graduate school	81	40.5
	Missing	7	3.5

Length ofpatient-physician relationship	≤1 year	97	48.5
	1–3 years	46	23
	3–5 years	11	5.5
	≥5 years	35	17.5
	Missing	11	5.5

**Table 3:** Factor coefficients of RPAD after varimax rotation.

Item	Factor		
	1	2	3
(1) Explain the clinical issues or the possible decisions	**0.752**	0.080	−0.091
(2) Discuss the uncertainty associated with the condition	**0.697**	0.059	0.163
(3) Clarification of agreement	**0.704**	0.100	0.281
(4) Recognize barriers to follow-through with treatment plan	−0.008	**0.742**	0.213
(5) Physician gives patient opportunity to ask questions and checks patient's understanding of the treatment plan	0.059	**0.756**	0.049
(6) The professional expression of the physician matches the patient's level of understanding	0.286	0.132	**0.532**
(7) Physician asks, “any question?”	0.047	−0.235	**0.731**
(8) Physician asks open-ended questions (i.e., does the patient have any other considerations or questions)	0.009	0.292	**0.603**
(9) Physician checks patient's understanding	0.354	**0.666**	−0.176

## Data Availability

The data used to support the findings of the study are available from the corresponding author upon request.
